# Disclosure experience in a convenience sample of quebec-born women living with HIV: a phenomenological study

**DOI:** 10.1186/1472-6874-12-37

**Published:** 2012-10-18

**Authors:** Geneviève Rouleau, José Côté, Chantal Cara

**Affiliations:** 1 Research Chair in Innovative Nursing Practices, Research Center of the Centre hospitalier de l’Université de Montréal, Montreal, Quebec, Canada; 2 Faculty of Nursing, Université de Montréal, Montreal, Quebec, Canada; 3 Center for Interdisciplinary Research in Rehabilitation, Montreal, Quebec, Canada; 4 CRCHUM, Hôtel-Dieu, 3840, St-Urbain Street, Pavillon Jeanne-Mance Site, 3rd floor, door 7-325, Montreal, Quebec, H2W 1T8, Canada

## Abstract

**Background:**

In Canada, there has been a considerable increase in the number of women infected with the human immunodeficiency virus (HIV). Within a stigmatized social context, disclosure of HIV positivity is still a prevailing concern among women. Little is known about the global understanding of how French-speaking, Quebec-born women living with HIV, live their serostatus disclosure experience. The aim of this qualitative study is to describe and understand the disclosure experience of these women.

**Methods:**

We conducted semi-structured interviews with seven women. A convenience sample of French-speaking, Quebec-born women was chosen because they all responded to the criteria of wishing to share their disclosure experience. The mean age of the participants was 46 years old (SD±12). They lived with HIV for an average of 10 years; time since diagnosis varied from 8 months to 23 years. Two out of four mothers had given birth to HIV positive children. Data analysis proposed by van Manen was performed to discover the essential themes of the experience.

**Results:**

Seven themes were identified to understand the experience of disclosure in women: 1) Respecting for self and confidants; 2) Feeling apprehension; 3) Exercising control to ensure protection; 4) Deliberately engaging in a process of disclosure/non-disclosure; 5) Exposing oneself to stigma and social exclusion; 6) Suffering internally; and 7) Benefitting from the positive effects of one’s decision. For these women, disclosing their HIV status meant: Living the ambivalence of a paradoxical process of revealing/concealing, in a state of profound suffering, exacerbated by stigma, while also being enriched by the benefits attained.

**Conclusions:**

Understanding the experience of disclosure in WLHIV is important to guide actions in the practice to support and accompany these women in their unique reality. Health professionals have to broaden their role and work on individual, interpersonal, inter-organizational and intersectoral levels. Mobilization of actors from different sectors would facilitate the implementation of pertinent and opportune interventions.

## Background

The number of women infected with the human immunodeficiency virus (HIV) grew considerably in Canada in the past decade. From 1985 to 1997, women accounted for 11% of positive HIV tests. By 2008, the rate had sprung to 26.2% [1]. Disclosing their serostatus to others–a condition often perceived negatively, identified with socially marginalized groups, and associated with sexually transmitted diseases and questionable behaviour [2,3]–is a major challenge for women living with HIV (WLHIV). In a stigma-impregnated culture [4,5], it is not surprising that disclosure is a complicated and difficult process. According to Sandelowski et al. [5], the fact of being a woman, the ability to bear children, and the possibility of infecting their offspring, all contribute to the unique experience of living with a stigmatized disease such as HIV infection. Even if the existing literature brushes a general picture of the phenomenon of disclosure in terms of patterns, recipients, frequency, consequences, reasons, reactions, and factors [6-13], little is known about the specific experience of French-speaking, Quebec-born WLHIV. Most of the existing studies have targeted a population from United States, Africa and Asia [4,13-21]. As it is recognized that disclosure is influenced by ethnicity and sociocultural context [9,21-26], it is not unreasonable to suspect that disclosure experience among French-speaking, Quebec-born women, belonging to a Canadian (Quebec/Montreal) society, might differ from the experience of disclosure in female populations in other countries and societies. The results of a qualitative study conducted in Montreal among WLHIV [26] pointed out differences in the experience of disclosure between Quebec-born women and those originally from Africa or Haiti. Quebec-born women perceive less HIV-related stigma and disclose their HIV serostatus more than participants of different ethnocultural origins. The latter are doubly stigmatized: the stigma against their ethnic culture is accentuated by the taboos within their own communities.

Various conditions can influence disclosure. These include the confidant’s ability to keep a secret, whether or not a climate of trust exists, a feeling of closeness and intimacy, the nature of the relationship (sexual or non-sexual), the consequences anticipated, one’s perception of being stigmatized, and prior disclosure experiences [8,20,27,28]. In light of these conditions, interpersonal relations are clearly central in the decision to disclose or not. HIV-positive persons must choose carefully who they reveal their serostatus to [27,29-31]. Moreover, it appears that there is a prior evaluation of the rewards and costs of disclosing to family and friends before disclosure occurs, which is supported by Consequence Theory [10,32]. This theoretical model proved useful in explaining disclosure in a study involving 125 HIV-positive women [10]. The principal rewards (benefits) for these WLHIV had more to do with the safety of others and their right to be informed than with oneself. The perceived costs (negative consequences) were in connection with the negative aspects of breaking the news, such as the fact of being reprimanded [10]. Disclosure occurs if the person perceives the benefits to outweigh the negative consequences and if it can thus avoid situations likely to cause harm [32].

Disclosing one’s serostatus or keeping it a secret carries both positive and negative consequences. Positive outcomes and reactions to disclosure such as support, comfort, help, and acceptance can be helpful and favourable [12,13]. However, after revealing their serostatus, some women have suffered adverse consequences such as violence, humiliation, rejection, and discrimination [6,7,19,33]. Though keeping a secret can be difficult to live with because it can lead to physical and emotional isolation and interfere with the establishment of trust and emotional closeness [5,34], it does allow focusing better on one’s present health needs without having to deal with people’s negative reactions.

Therefore, both disclosure and concealment are experienced as a conflict, a dilemma, or a paradox [16,35,36]. Parse [37,38] defined a paradox as rhythms lived simultaneously rather than in opposition or as a dissociated dilemma. In other words, disclosure and secrecy are frequently experienced at the same time and not as two dichotomous entities.

Against this background, we undertook a qualitative study to describe the disclosure experience of French-speaking, Quebec-born WLHIV. The study was geared to identifying the essence of disclosure (gain an understanding of the whole) from a phenomenological viewpoint without targeting specific components of the experience beforehand (e.g., recipients, reactions, consequences) but instead allowing the meaning simply to emerge.

## Methods

The hermeneutic phenomenological method proposed by van Manen [39] was used to study the lived experiences of disclosure as perceived by WLHIV and to arrive at the essence of the phenomenon. This type of qualitative study serves to extract the profound and rich significance of a phenomenon [40]. As recommended in phenomenology, bracketing was used throughout the research process to describe the principal investigator’s beliefs, preconceptions, biases, and knowledge [39].

### Ethical considerations

The project was approved by the Research Ethics Review Board of the *Centre hospitalier de l’Université de Montréal*. Consent was obtained after a full verbal and written explanation of the study was given to participants and after they were informed they could withdraw from the study at any time. Additionally, the participants were assured that every measure would be taken to maintain confidentiality throughout the research to protect their identity [41], including the use of pseudonyms and the modification of data in the interviews (e.g., workplace, physician’s name).

### Setting and participants

This study was conducted among French-speaking, Quebec-born WLHIV. A convenience sample of seven participants was chosen and they all responded to the criterion of wishing to share their experience of disclosure. Women with cognitive impairments that prevented them from expressing their experience, those who kept their health situation completely secret (“total” concealment), and active intravenous drug users were excluded. Study participants were recruited from two Montreal healthcare facilities: a teaching and research centre of a large metropolitan university hospital that cared for people living with HIV (PLHIV), and a medical clinic specialized in HIV care and follow-up. Eight women interested in the research were identified by nurses and physicians at both sites and were put into contact with the principal investigator. Seven of the women participated in the study; one was absent at time of interview owing to psychosocial problems.

Participants ranged in age from 32 to 64 years, the average being 46 (SD±12). All were White and Quebec-born and all lived in Montreal and surrounding areas. Some had learned of their HIV-positive status eight months earlier, while others had been living with HIV for more than 20 years (10 years on average). Four were single and three lived with a partner. Four women had children (one to three) ranging in age from 10 to 36 years. Two of the four mothers had given birth to HIV-positive children. Four of the women were employed, two were retired and did volunteer work, and one was working her way through school. Three participants had a university degree, three, a college degree, and one, a high school diploma. In the year prior to the beginning of the study, four participants reported income from CAN$25,000 to CAN$34,000, two fell below this range and one, above it.

### Data collection

The principal investigator conducted a semi-structured interview lasting from 45 to 70 minutes with each participant. As pointed out by Paley (1997), the aim of phenomenological research is to get at the essential structure of a phenomenon, its rich and profound meaning, regardless of sample size. Data redundancy was attained [42] with a sample of seven participants. In other words, the rich and profound data to emerge from the experiences of the participants allowed reaching saturation after completion of the seven interviews conducted. Interviews were recorded and then transcribed. With the aid of an interview guide, women were encouraged to share their personal disclosure/non-disclosure experiences, including what they thought and felt at the time and the reactions of their confidants. The questions used are presented. We felt that letting participants express themselves freely in their own words would allow a deeper exploration and lead to a richer description and understanding of their experiences.

Interview guide

Can you tell me about a specific situation where you disclosed to someone that you were HIV-positive?

What were you feeling before making the disclosure?

What was going through your mind before making the disclosure?

How did you break the news?

What were you feeling while making the disclosure?

What was going through your mind while making the disclosure?

Can you describe how the person reacted to the news?

What did you feel after making the disclosure?

What went through your mind after making the disclosure?

Can you tell me about a specific situation where you chose not to disclose to someone that you were HIV-positive?

How did the fact that you did not disclose your status make you feel? What were you feeling when you chose not to make the disclosure?

What was going through your mind when you chose not to disclose your status?

What meaning does your disclosure experience have for you? What does disclosure mean to you?

### Data analysis

Two research activities proposed by van Manen [39] were used to guide data analysis: reflection and writing (description). Reflection consisted of discovering themes within the experiences using three approaches: 1) holistic, 2) selective, and 3) detailed. These allowed gaining an understanding of the meaning of disclosure in general (holistic) arriving at meaningful descriptions of the experience of disclosure (selective), and garnering details of the lived experiences of the participating WLHIV by sifting through the interview transcripts [39]. Several meaning units were identified, which yielded a series of sub-themes (n=29), from which emerged a few major themes (n=7). Writing reflected the interpretation and thoughts of the researcher and her openness to all forms of language. The interviews were read several times by the principal investigator and by one of the co-investigators. The dynamic and iterative interpretative process–going back and forth throughout the stages of analysis and the repeated readings of the interviews–is what would allow the essence to emerge.

### Trustworthiness of the study

Credibility is, according to Lincoln and Guba [43], one of the most important criteria for establishing trustworthiness. Results are credible when the phenomenon under study is recognized by participants and experts and it reflects their personal experience [43,44]. The authenticity criterion refers to the fact that results must be in line with or reflect the experiences described and lived by participants [44]. In the aim of meeting these criteria, the principal investigator: used bracketing; read the interviews many times over; went back and forth between data collection and analysis; reached data saturation; used peer reviewing and held debriefing sessions with the two research directors (supervisors) regarding the data collected, analysis and interpretation.

## Results

**Figure 1:**
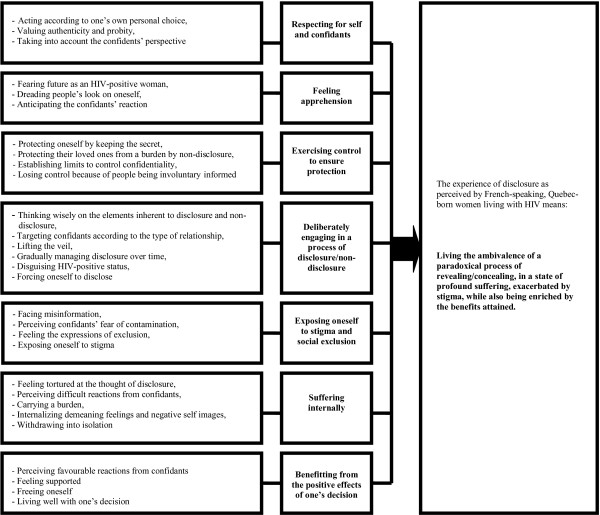
**Results.** 29 sub-themes were identified, from which seven major themes emerged: these allowed the discovery of the essence.

Analysis of the seven interviews yielded 29 sub-themes from which seven major themes emerged. These allowed arriving at a better description and understanding of the essence of the experience of disclosure (Figure 1).

### Theme 1: Respecting for self and confidants

This first theme illustrated the importance for WLHIV of maintaining their self-respect throughout their disclosure experience and of respecting their confidants. For these women, disclosure is a matter of personal choice. Having the freedom to choose to disclose or not respected their uniqueness and the distinctive characteristics of each lived experience. Making a decision (disclosure/non-disclosure) that is considered desirable and acceptable represented, for women, a way of maintaining self-respect. Claire, age 46 and HIV-positive for more than two years, illustrated the importance of her personal choice as follows:

"I think this is something very personal for everyone. Those who decide to carry a banner and say, “Well, OK, I’ll say it out openly and […], I would tell them that, ultimately, it’s their choice: “It’s your call”. I also think we must respect people who decide otherwise."

The participants also valued their own honesty, sincerity, and truthfulness, which meant that they felt the need to disclose their secret and share their experience and to be themselves in spite of their health condition. However, there was a difference between what was desired and what was feasible, that is, between disclosure and concealment. According to Nicole, age 32 and HIV-positive for almost two years, keeping her serostatus a secret meant compromising her authenticity: “*Well, it’s hard. It’s hard because I don’t feel completely honest.”* While respecting their own values, participants showed empathic respect towards their confidants, who required time for the news to sink in. Kate, 60 years old and HIV-positive for 20 years, expressed it as follows: “*You have to give them time to get used to the idea [HIV-positive status].”*

### Theme 2: Feeling apprehension

The women in the study felt apprehensive about disclosing and feared looking ahead to an unknown future, imagining every possible scenario. In addition to anticipating the difficulty of facing future disclosures, most participants talked of the uncertainty of an unknown and unfamiliar health status and the fear of ending up alone and of never finding someone who would accept and want them. Melissa, age 36 and diagnosed HIV-positive eight months earlier, expressed her apprehension about revealing her serostatus to a potential partner as follows:

"So that’s that. Time will tell, right? If I look into the future, do you think I can imagine myself with someone? How would I tell him? When would I tell him? Before? After? At the very beginning?"

The women were also distrustful and suspicious that others might figure out they were HIV-positive at a glance. In other words, they dreaded the way people might look at them. Here is how Suzanne, 49 years old and HIV-positive for 16 years, put it: *“Every time someone looked at me or my son, I suspected the person was thinking this or that. It’s wrong to think his way, you know.”*

After entrusting someone with their secret, the women awaited the reaction of their confidant. They were unsure what it would be until the moment of disclosure when they were finally faced with it. There was no way of knowing beforehand what it would be: “*Ultimately you say, um…well, maybe this is how they should react, but you know deep down that there’s no guarantee. You don’t really know how people are going to react*” (Claire, 46 years old, HIV-positive for more than two years).

Facing situations where reactions were uncertain or were expected to be negative was a difficult experience.

### Theme 3: Exercising control to ensure protection

Control was exercised through non-disclosure and by setting limits. By limiting the information shared or limiting the number of persons with whom the information was shared, the women protected not only themselves from possible threats but also their loved ones. Ultimately, control could not be exerted in optimal fashion; that is, protection from negative consequences was seriously compromised when one’s HIV-positive status was unintentionally disclosed. Several participants maintained control over their information by keeping their status a secret because this was considered a good way of protecting oneself from threats, danger and other negative consequences or impacts of disclosure, such as stigma, rejection and discrimination. Jenny, 64 years old and living with HIV for 23 years, explained it in the following way:

"Maybe I should have disclosed my situation in the beginning. But I was working full-time then… I didn’t want to suffer rejection and I didn’t want everyone talking about it in the office. Plus, I was afraid of losing my job, you know… So, I kept quiet about it."

Moreover, women exerted control by deciding not to disclose their status to their loved ones and thus spare them from suffering distress and carrying a burden. The experience of causing such anxiety is illustrated in the following account:

"I tell myself I don’t want people to pity me. The moment you have a little cold, it’s: “Are you OK?” You know? (laughs) That’s why I prefer people don’t worry about me. You know, I’m fine. Why should I stress people out? (Suzanne, 49 years old, HIV-positive for 16 years)"

All the women tended to restrict or filter the information on their health status with certain people and under certain circumstances in order to protect themselves. However, certain confidants also wanted to impose their own limits in order to reduce disclosure. This was the case for Sarah, age 37 and HIV-positive for more than seven years: “*Well, he himself* [her husband] *did not want to tell his family*”. Her husband wanted to protect the couple and his family from the risks associated with disclosure. In this way, non-disclosure prevented confidential information from spreading and ensured control of the situation.

Certain women felt a loss of control when people around them came to know of their HIV-positive status involuntarily. In some cases, they realized that their confidants needed to talk to someone else and share the secret. In one case, the woman’s serostatus was discovered at the birth of her HIV-positive child. In another, medication in the house gave the kids away: “*They* [her kids] *were curious*. *They were on the computer and looked up the name of one of the drugs. That’s how they figured out I had it* [HIV]” (Suzanne, 49 years old, HIV-positive for 16 years).

### Theme 4: Deliberately engaging in a process of disclosure/non-disclosure

All the WLHIV reported preparing themselves in some way and asking themselves some hard questions, which testified to their engagement in a process of disclosure/concealment. It involved a careful analysis of the aspects associated with revealing or concealing one’s serostatus, as expressed by Sarah (37 years old, HIV-positive for more than seven years): “*It’s always, like, who am I dealing with? What can I say? What is it I can’t disclose? How far can I go?”*

Disclosure as perceived by these women also meant establishing a set of “criteria” with which to assess one’s relationship with a potential news recipient. These included type of personality, closeness, ability to keep a secret, level of trust, and others. For example, Melissa (36 years old, HIV-positive for eight months) explained:

"My, my, my brother has, has a, has a girlfriend. Well she, she doesn’t know about it. But everyone else does. Because she’s too, she’s too loose lipped. She could blab it all out anytime, you know? I can’t trust her."

This underlined the reflective, evaluative, and analytical nature that characterized, in part, the commitment taken in a process of disclosure/concealment. This process led to action when the women decided to lift the veil and reveal their HIV status. The participants gave detailed examples of their lived experiences of disclosure specifying who the confidant was, the manner in which they revealed their secret, the circumstances surrounding the event, and the place where it happened. Jenny (64 years old, HIV-positive for 23 years) described a situation of disclosure in a propitious setting:

"[…]I started talking without ever thinking I was going to tell the person about it. Like… with my sister, we were sitting outside on the swing and we started talking. All of a sudden I felt like telling her. So I did."

All of the participants also concealed their serostatus by masking their HIV-related symptoms. For instance, they gave vague explanations for hospital stays or for their deteriorating state of health; they allowed uncertainties about their health to persist; and they hid their medication. They might even lie about their health if they felt trapped: “*In fact, the insurance agent wasn’t too smart that way; he asked all the confidential questions in front of my mother. So, uhm, as far as my health was concerned I had to lie*.” (32-year-old woman, HIV-positive for nearly two years).

The disclosure experience lived by the WLHIV in our study was described and understood as a dynamic and evolving process. It was lived gradually, one step at a time. One of the participants living with HIV for more than 20 years kept her serostatus a secret for ten years before deciding to talk about it.

Finally, the participants sometimes felt compelled to reveal their health status, for instance, by their sense of duty and responsibility towards second or third parties at risk of being infected, as in the event of sexual relations, injury or an open wound. Furthermore, in cases of vertical transmission, mothers felt compelled to tell their children they were HIV-positive; they felt their children had the right to be informed: *“He has to know. He has a right to know he’s HIV-positive”* (Kate, 60 years old, HIV-positive for 20 years).

### Theme 5: Exposing oneself to stigma and social exclusion

Being exposed to a stigmatizing environment that encourages social exclusion was part of the participants’ disturbing reality. In fact, the sociocultural environment was rather unfavourable to HIV-positive people, marked as it was by prejudice, preconceptions and misinformation as well as fear of infection and a tendency to social exclusion. All the participants mentioned the lack of HIV-related information in the general population and also among health professionals. For Kate (60 years old, HIV-positive for 20 years), this lack of knowledge contributed to stigma: *“Otherwise, we keep the same prejudices. If we have no information… our conceptions don’t evolve, nor does our perception of the infection.”* Offensive tones, remarks or statements could also be a consequence of misinformation that exposed participants to stigma and social exclusion.

The majority of the participants indicated that perceiving fear of contamination in people was inherent to disclosure given the transmissible nature of the infection. The perceptions and interpretations of the women in our study arose from their own experiences: people avoiding to share food with them, refraining from comforting them when crying for fear of contamination from tears, or withholding a handshake as a way of limiting physical contact. Kate (60 years old, HIV-positive for 20 years) expressed it eloquently:

"They’re so scared if we’re around and if we’re eating… at the same table… “Don’t touch that fork!” You know, it was, like, “Oh, OK”. Fact is, you’re not hungry anymore, you don’t wanna eat there no more, you don’t wanna do nothing. I mean, my brother would go: “Well, don’t use the same glass.” “No, no”. When I went to their place I took along my own glass with me (laughter)."

Strongly upheld by all the participants was the exclusion and rejection from close relatives or friends and from health professionals who had no wish to keep the HIV-positive women under their care. Jenny (64 years old, HIV-positive for 23 years) bore witness to this in the following excerpt:

"I have felt rejection, well… I called my dentist, my doctor, um, my family doctor. They didn’t want anymore… they saw me, but they told me they didn’t want to see me anymore, that it wasn’t them I should see. The dentist didn’t want to treat me anymore. This was a blow."

After disclosure, some friendships and romantic relationships came to an end. There was a gradual separation that finally ended with the absence of news. Inevitably, the end of a relationship marked by a feeling of exclusion. The taboos, prejudices, and negative judgments surrounding HIV infection emphasized stigma. Melissa (36 years old, HIV-positive for eight months) put it like this:

"…but, he caught it by not protecting himself, by screwing around, you know. It’s not the same thing. Like, that’s what’s disgraceful. Those are the taboos we were just talking about before: “You are sick because you weren’t careful”, you know. You touch on many taboos, you know, when you tell someone this. You touch sexuality, death, and disease. People don’t want that, you know."

Regarding disclosure, one of the participants, who visited a community organization for WLHIV, noticed a difference between White women and Black women. Although this perception did not emerge from all the participants, some perceived that the experience of disclosure could differ from one culture to another, as expressed by Jenny (64 years old, HIV-positive for 23 years):

"…but what I learned here about disclosure is, that other, other nationalities don’t have it as easy as us, I think. They are still…, because they don’t want to tell their families […] It’s not the same as for us. It is uhm … more hidden. They don’t have to say it. The mentality is not the same at all, at all."

Therefore, even if the participants were born in a North American context, which is socially more favourable, they were not free from social stigma, as they could be affected by it directly or indirectly. The experience of being different, rejected, or “excluded” marked their reality of being HIV-positive.

### Theme 6: Suffering internally

For the participants, disclosure was an extremely painful experience and they did not always feel capable of going through with it. This accounted in part for their inner suffering. In fact, for some participants such as Suzanne (49 years old, HIV-positive for 16 years), disclosure was even perceived as torture: *“I didn’t like that at all. I would just as soon have my neck wrung instead… you know. I would. When there is really nothing else that can be done, then I talk.”* Another source of suffering, unanimously shared by all participants, was the reactions from confidants, as disclosure was frequently an unexpected, confrontational, and emotional event. These reactions covered shock, sadness, anger, rage, and discomfort. Unquestionably, they were “absorbed” by the participants and thus increased their inner suffering.

A participant (60 years old, HIV-positive for 20 years) shared her painful experience of a double disclosure when she broke the news to her son that they were both HIV-positive:

"It was a difficult trip back, you know. The anger, the rage, I… you know… “I didn’t do anything to get that. Why me? It’s your fault”. Yes, it was my fault. So, yes, we cried together. Then we went through all the emotions together. Then he made me pay, and made me pay dearly. He was nasty to me."

"Well, this anxiety. You tell yourself, dear God, how do you tell your child that you transmitted to him something that he will have for the rest of his life, that cannot be cured? There are no words to explain how you feel faced with something like that, you know. Can you imagine telling your child: “Look, I am the one responsible, I am guilty?"

Most of the participants also indicated that they suffered from the burden of keeping a secret–the corrosive silence–or from the guilt of having transmitted the virus to their children. Sarah (37 years old, HIV-positive for more than seven years) had this to say about the former: *“It’s a heavy weight to bear. It’s a silence that eats away at you on the inside.”*

Several participants described the shame of being HIV-positive, the poor image they had of themselves, and their feeling of worthlessness. Concretely, the participants did not feel like women anymore, they felt dirty, they felt like a “rag”, they did not feel like good persons on account of being infected with the virus:

"Ah well, for sure, you don’t think of yourself uhm… you know what I mean, it’s… you don’t think very highly of yourself as a person (giggle). It’s uhm… It’s, it’s hard. It’s hard on your ego, you know, you tell yourself that… that you contracted HIV uhm. I feel like a rag for having come down with it, you know."

Without exception, every woman interviewed experienced exclusion and solitude, which meant isolating themselves from the rest of the world. Some broke off either totally or partially with their social contacts, while others, like Claire (46 years old, HIV-positive for more than two years), attested to the danger of isolation that threatened them: “*Well, that’s the danger… people with the virus… you have a tendency to withdraw into yourself and never see anyone. And that is very dangerous, I think, especially for women. I think women have a great tendency to… withdraw*.” As for Melissa (36 years old, HIV-positive for eight months), her suffering was perceived through feelings of solitude: “*Fact is, what is really sad is that we all live with our little solitude inside. That, that’s the worst part about it! It’s almost sadder than the fact of being HIV-positive.”*

### Theme 7: Benefitting from the positive effects of one’s decision

Fortunately, disclosure/non-disclosure was also lived as a positive experience. Once their situation revealed, participants indicated that they lived experiences of love, surprise, encouragement, compassion, and acceptance. For Melissa, her family’s love seemed essential for accepting her situation: “*It’s true that family, well, they have a tie, a love tie with you, so … whether you protected yourself or not, or whether you knowingly made a mistake or not, they love you anyway.”*

Feeling supported was the sub-theme most frequently mentioned by the women in our study and it is essential to illustrate the benefits of disclosure. It was described in different ways depending on each experience. Support was perceived as a wish or expectation, as a reason for disclosure or as a consequence of it. Indeed, women seemed to reveal their serostatus in order to obtain support, which corresponded to the reason for the disclosure. When participants derived support from disclosure, it became a positive consequence. The positive effects of support facilitated the adaptation of participants to their reality and it helped them through hardship. Claire expressed the importance of support in her life through the following words: “*Well, I have had lots of support, and… in this situation, I think it’s essential.”* Participants felt supported by different people, especially family members, their partners, friends, health professionals–through the expertise, follow-up and information provided–and other HIV-positive people who had lived similar experiences in spite of the uniqueness of each situation.

Revealing their HIV-positive status allowed the women to express what they were experiencing and feeling. For example, voicing how they felt gave them a sense of relief:

"It’s a relief. Not just for you. Being um… able to talk openly. You can do so… when things are going well for you and when they’re not. You can express what it is you’re experiencing. To have someone you can trust, that’s important. (Kate, 60 years old, HIV-positive for 20 years)"

Unburdening oneself allowed sharing both the experience locked up inside and how one felt regarding the benefits and positive consequences of such a decision.

Whether the decision was to disclose their status or keep it a secret, some participants reported living well with their choice, thus attesting to a feeling of well-being and comfort. As Suzanne (49 years old, HIV-positive for 16 years) put it: *“I feel well anyway because I don’t want to complicate my life. I feel well not talking about it*.*”*

Though most of the experiences of disclosure described by the participants stirred up sad memories, the benefits that they derived from disclosure were of great importance to them.

### Essence of the phenomenon

The seven themes presented above contributed to the emergence of the essence of the phenomenon of disclosure by WLHIV. For these women, disclosure of their HIV-positive status meant living the ambivalence of a paradoxical process of revealing/concealing, in a state of profound suffering, exacerbated by stigma, while also being enriched by the benefits attained. In this section, this essence will be rendered explicit and the contribution of each of the themes to this end will be demonstrated.

The ambivalence of the paradox distinctly defined the experience of disclosure lived by these HIV-positive women. For example, respecting for self and confidant at the same time meant striking a balance between the two. Ambivalence was expressed also by feelings of apprehension, experienced as friction, uncertainty and fear of the consequences of disclosure, an array of simultaneous disturbing thoughts. On the other hand, there was also control that the HIV-positive women tried to maintain in order to protect themselves against the potential risks of disclosure, such as stigma, rejection or judgment. Adding to the suffering and to the paradoxical aspect of disclosure was the stigmatizing social context, which remained, for the most part, an uncontrollable element. One of the facts that emerged was that most of the participants were marked by this dominant stigma despite having an advantageous social status, not originating from a country where HIV is endemic.

The process of revealing/concealing allowed us to describe and gain a better understanding of the essence of the phenomenon especially in light of deliberate and voluntary engagement in the disclosure/non-disclosure process. Thus, careful deliberation, effectively targeting a confidant, lifting the veil on a secret, managing gradual disclosure, keeping one’s serostatus hidden and forcing oneself to disclose, all speak of the rhythmic balance at the heart of this process.

Finally, the experience of disclosure allowed women to benefit from the positive consequences of their decision and to appreciate those consequences. In a way, gaining from these benefits was the flipside of suffering and stigma, thus emphasizing even further the paradoxical ambivalence that pervaded the process. The benefits obtained or the expectation of obtaining benefits co-existed with the apprehension of negative consequences, control to ensure protection, suffering, and stigma.

## Discussion

The aim of our qualitative study was to describe and arrive at a better understanding of the experience of serostatus disclosure as perceived by French-speaking, Quebec-born WLHIV. The study’s main contribution to the field was to extract the essence or meaning of disclosure from the participants’ *verbatim* transcripts. In other words, the unique experience of each of the seven WLHIV yielded rich and exhaustive data–29 sub-themes and seven themes–that allowed gaining a holistic understanding of the profound and complex nature of disclosure.

Our results substantiate the complexity of the experience of disclosure in light of the paradoxes lived simultaneously and not as two separate or opposite events. The results support the concept of paradox as defined under Parse’s theory of nursing [37,38,45]. Indeed, revealing (disclosing)/concealing (not disclosing) represented the paradoxical process of talking about something and, at the same time, hiding a part of reality. Another paradox was often experienced as well, that of “enabling/limiting”. This occurred when obtaining benefits (support, relief) and placing restrictions (risk, suffering) were lived simultaneously [37,38].

The first theme, respecting for self and confidants, broadens what we already know by helping us understand the disclosure experience in terms of respect for oneself and others.

The second theme, feeling apprehension, supports several authors in the literature [30,46-50] who identified the anticipation of reactions, along with the future, life expectancy and possible other disclosures, as a source of uncertainty and stress for HIV-positive women.

Exercising control to ensure protection, the third theme, supports what some authors have written about the reasons for not disclosing [3,7,29,30,47,50-53]. Essentially, it is to prevent a host of negative impacts, including fear of rejection, stigma, ostracism, social exclusion, discrimination and breach of confidentiality, but it is also to avoid worrying others [14,50,52,54,55].

The fourth theme is described as engaging voluntarily in a paradoxical process in which situations of disclosure and concealment are lived simultaneously and supported by reflection and are the subject of profound deliberation. This finding extends the literature if we consider the definition of this theme, which is well understood throughout its entire process by the importance of these six sub-themes: 1-thinking wisely on the elements inherent to disclosure and non-disclosure; 2- targeting confidants according to the type of relationship; 3-lifting the veil; 4-gradually managing disclosure over time; 5- disguising HIV-positive status and; 6-forcing oneself to disclose. All of these sub-themes are consistent with numerous works in the literature regarding the choice of confidant, the right moment and way to disclose, the circumstances surrounding disclosure, and the moral obligation to disclose [5,8,9,13,18,30,56]. In sum, it is fair to say that most of the participants experienced their disclosure freely and openly while exercising control over the process.

The fifth theme, exposing oneself to stigma and social exclusion, was lived by all the women in our study. This theme is well supported by the literature [2,3,5,35,53]. In accordance with Greene et al. [30], the results of our study lead us to believe that being exposed to social stigma and exclusion adds to the complexity of HIV disclosure. Furthermore, we agree with Parker and Aggleton [57] who conceptualize stigmatization as an “uncontrollable” and “external” process that reinforces social inequalities and existing relations of power. In light of our results and those of Parker and Aggleton [57], it seems reasonable to conceive stigma well beyond the individual level and consider it instead as a social process, in which interventions could be aimed at transforming the political, cultural and social setting. The women in our study all mention the existence in their society of HIV-related prejudice, stereotypes, and taboos. It is inherent to their lives to be exposed to a stigmatizing social context. This is a painful reality out of one’s control that increases the complexity of disclosure.

The experience of disclosure as perceived by these women is marked by inner suffering (sixth theme) due to the difficulty–if not “torture”–of revealing their HIV-positive status, the reactions of their confidants, the burden of their secret, isolation, a negative self-image, apprehension, and stigma. All of these aspects define the suffering experienced by WLHIV, which corroborates the literature on the burden of secrecy [5] that can lead to isolation [35]. This is an inner dimension of stigma [58,59] that touches self-esteem [60].

Although disclosure can be a difficult experience, benefitting from the positive effects of such a decision (seventh theme) allows us to understand the possibility for HIV-positive women to live well with the decision of revealing/concealing. This constitutes a new angle (living well with the decision) from which to describe the phenomenon of disclosure. Our results agree with those previously reported by other authors [10,18,61,62] on the positive consequences, benefits or rewards of disclosure such as catharsis and support from the confidant*.* In conclusion, the added value of our results is that they serve to illustrate and describe the benefits within a paradoxical process that allows reaching a deeper understanding of the meaning of disclosure.

The study has various limitations. Although van Manen [39] and Benner [42] recommended carrying out a second interview in order to validate the researcher’s interpretation and thus increase the authenticity of results, we conducted only one interview owing to the delicate and demanding nature of disclosure and to the difficulties encountered in setting up the first meeting. However, various measures were taken to meet the scientific criteria of authenticity [44] and credibility [43,63], as mentioned earlier in the Methods section.

## Conclusion

Actions in clinical practice are geared towards supporting and nurturing WLHIV. We believe that healthcare professionals must broaden their role and work on various levels: individual, interpersonal, inter-organizational and intersectoral. The mobilization of actors, professionals, and caregivers from different sectors would be useful to gaining a better understanding of the complex nature of the phenomenon of disclosure. This in turn could facilitate the implementation of pertinent and imperative interventions. Such interventions are required not only to reduce the suffering and stigma of these women but also to achieve and reinforce the potential benefits of disclosure/non-disclosure.

## Abbreviations

HIV: Human immunodeficiency virus; WLHIV: Women living with HIV; PLHIV: People living with HIV.

## Competing interests

The authors declare that they have no competing interests.

## Authors' contributions

GR carried out the entire study, participating in the design, conducting interviews, analyzing and interpreting the data, and drafting the manuscript. JC supervised the entire study and was involved in interpreting the data and drafting the manuscript. CC carried out the study, supervised data analysis and interpretation, and drafted the manuscript. All authors read and approved the manuscript.

## Authors’ information

GR, MSc, RN, is research coordinator of the Research Chair in Innovative Nursing Practices at the Research Center of the *Centre hospitalier de l’Université de Montréal (CRCHUM)*, Montreal, Quebec, Canada. JC, PhD, RN, is holder of the Research Chair in Innovative Nursing Practices at the CRCHUM and full professor at the Faculty of Nursing of the *Université de Montréal*, Montreal, Quebec, Canada. CC, PhD, RN, is full professor and associate dean of graduate studies at the Faculty of Nursing of the *Université de Montréal*, and researcher at the Center for Interdisciplinary Research in Rehabilitation, Montreal, Quebec, Canada.

## Pre-publication history

The pre-publication history for this paper can be accessed here:

http://www.biomedcentral.com/1472-6874/12/37/prepub
